# A Four-Wave Cross-Lagged Study of Exposure to Violent Contexts, Cognitive Distortions, and School Bullying during Adolescence

**DOI:** 10.3390/ijerph21070883

**Published:** 2024-07-07

**Authors:** Mirella Dragone, Dario Bacchini, Concetta Esposito, Gaetana Affuso, Grazia De Angelis, Fabrizio Stasolla, Raffaele De Luca Picione

**Affiliations:** 1 Faculty of Law, Giustino Fortunato University, 82100 Benevento, Italy; m.dragone@unifortunato.eu (M.D.); f.stasolla@unifortunato.eu (F.S.); r.delucapicione@unifortunato.eu (R.D.L.P.); 2 Department of Humanistic Studies, University of Naples “Federico II”, 80133 Napoli, Italy; dario.bacchini@unina.it; 3 Department of Psychology, University of Campania “Luigi Vanvitelli”, 81100 Caserta, Italy; gaetana.affuso@unicampania.it; 4 Department of Psychology and Educational Sciences, Pegaso University, 80143 Napoli, Italy; grazia.deangelis@unipegaso.it

**Keywords:** adolescence, bullying perpetration, community violence exposure, domestic violence exposure, self-serving cognitive distortions

## Abstract

School bullying represents a widespread expression of violence in the peer context. Guided by the social-ecological model, this study investigated the longitudinal and transactional pathways linking domestic and neighborhood/community violence exposure (through direct victimization and witnessing), self-serving cognitive distortions (CDs), and school bullying perpetration. Furthermore, consistent with the previous literature, we tested the cognitive desensitization process that could develop in response to chronically violent contexts. Two four-wave cross-lagged panel mediation models were tested in a sample of 778 high school students (28.1% males; *M_age_* [Time 1; T1] = 14.20, *SD* = 0.58). The results showed differential effects of multiple contexts and forms of violence exposure, with domestic violence victimization and community violence witnessing being associated with self-serving CDs and bullying perpetration over time. Moreover, significant associations between CDs and bullying perpetration over time were found, with bidirectional effects for each of these longitudinal patterns. Finally, self-serving CDs significantly mediated the relationships between both domestic violence victimization and community violence witnessing and school bullying perpetration. These findings highlight the need to consider school bullying as a social phenomenon stemming from a complex and bidirectional interplay between individuals and the environments they inhabit, confirming a basic postulate that “violence breeds violence”.

## 1. Introduction

School bullying is one of the major social problems affecting children and adolescents in all parts of the world [1]. This subtype of aggressive, intentional behavior carried out by a group or an individual repeatedly and over time against a victim who cannot easily defend him- or herself [2] has been commonly recognized as a widespread expression of violence in the peer context [3]. A recent report published by the United Nations Educational, Scientific, and Cultural Organization [4] revealed that more than 30% of the world’s students have been victims of bullying, bringing deleterious, immediate, mid- and long-term consequences in both the educational and mental health domains [3]. From a developmental perspective, such behaviors peak during middle school years (i.e., 12–15 years) and tend to decrease by the end of high school [5], assuming different forms across age groups [3]. Adolescence represents a fertile ground for bullying involvement due to biological, cognitive, emotional, and social changes occurring during this developmental stage [6]. This period is also marked by a growing need for popularity and dominance, which often motivates socially coercive behaviors (e.g., bullying; [7]) viewed as a deliberate strategy to re-establish social status and dominance when youth enter a new peer group [8].

In the framework of Bronfenbrenner’s [9] social-ecological model, school bullying has been investigated in light of several individual and contextual protective and risk factors [10]. Such a model focuses on understanding how individual characteristics interact with environmental contexts or systems to promote or prevent victimization and perpetration [1,10], assuming that bullying victims and perpetrators are part of the complex, interrelated multiple system levels that shape the individual—that is, micro-, meso-, exo-, macro-, and chronosystem levels [11].

Taking this framework into account [9], the current study extends upon a previous one [12] by incorporating an additional cohort of adolescents, new measures, and a longer period of assessment into the research design. More specifically, the main goal and the novelty of the present study was to investigate, over four years, in a high-risk urban area in Southern Italy where organized crime subculture is highly rooted [13], the longitudinal and simultaneous pathways linking youth’s violence exposure across family and neighborhood/community microsystems, respectively, as a victim and/or as a witness, as well as individual pro-violence moral cognitions (represented here by self-serving cognitive distortions—hereinafter CDs) [14], with school bullying perpetration.

To date, most studies evaluating the differential effects of multiple contexts (i.e., the family and the neighborhood/community) and multiple forms (i.e., through witnessing and direct victimization) of violence exposure in promoting bullying perpetration have raised conflicting findings, and the research on the potential mechanisms involved in the cycle of transmission of violence for youths growing up in violent contexts needs to be further explored.

In this regard, though there is strong evidence that exposure to both domestic (e.g., [15,16,17,18]) and community violence (e.g., [19,20,21]) and self-serving CDs [22,23,24] are associated with externalizing behaviors (i.e., aggression, conduct problems, delinquency), only a few studies, most of them cross-sectionals, have systematically examined how bullying behavior (as a specific subtype of aggression) is influenced by experiences of domestic (e.g., [25,26,27,28,29]) and community violence exposure (e.g., [12,30,31,32,33,34]). Moreover, the associations between violence exposure and moral CDs [12], according to Gibbs’ model, have been poorly investigated, as has the question of how such pro-violence moral cognitions are related to the involvement in bullying behavior [35].

Finally, consistent with the previous literature [36,37,38,39], this study sought to test the cognitive desensitization process that could develop in response to chronically violent contexts, by considering moral CDs as indicative of cognitive desensitization that would occur after repeated experiences of violence within the family and community. In this regard, most of the evidence comes from investigating the mediating role of acceptance of violence cognitions or biased social information processing between exposure to violence and aggressive behavior [40,41,42]; however, to date, the examination of the environmental precursors of moral attitudes underlying externalizing behavior in adolescence, as bullying, should be further investigated [12,43].

### 1.1. Exposure to Violence within the Family and Community as a Social-Environmental Risk Factor for School Bullying Perpetration

According to the ecological model applied to bullying [44,45,46], understanding the factors that promote bullying behaviors requires a close examination of the experiences youth have daily in the environments they inhabit outside of school, in both the more immediate or proximal (i.e., family) and distal (i.e., neighborhood/community) microsystems with which youths have direct interactions [1].

During adolescence, violence exposure is one of the experiences that reaches a dramatic peak [47,48,49]. This exposure can take place across multiple, sometimes overlapping contexts or settings (i.e., the concept of “poly-victimization”; [50]) that refer to the multiple ecological levels of the microsystem. Also, it can assume multiple forms, including direct victimization and witnessing without being directly involved.

Some studies have suggested a sort of equifinality of violence exposure across different contexts, which seems to eventuate in the same outcomes, whereas other studies have suggested a differential effect of violence exposure on developmental outcomes in the function of the involved microsystem. This may be due to the differential proximity to the child within the hierarchically ordered social ecology [13]. More specifically, some studies [38,51,52] suggested a stronger impact of violence exposure in the family than in other settings, revealing that domestic violence exposure was a more robust predictor of adjustment problems (e.g., anxiety, depression, aggression, and antisocial conducts) than exposure to community violence. However, other studies [15,19,53] found that community violence has a unique role in predicting several externalizing behaviors accounting for other sources of violence exposure.

Another key issue concerns the differential effects of witnessing or victimization experiences that appear to affect child development differently [54]. Overall, witnessing violence has been shown to be linked to externalizing behaviors (e.g., [55]) mainly through a social learning process that leads to the acquisition of deviant social information patterns, which in turn increases the likelihood of aggressive behavior (e.g., [33,53,56,57]). Conversely, direct victimization appeared to be more strongly associated with the development of internalizing symptoms (e.g., [58]) through impairments in emotional self-regulation that compromise the more general ability of an individual’s adaptive behavior [33]. Other studies have found no significant differences in the specific outcomes associated with witnessing and victimization experiences [59].

Referring to school bullying, although most of the research has highlighted that violence exposure within the family and community places youths at great risk for negative peer experiences such as bullying perpetration, the findings vary considerably across studies.

As regards the family context, a previous review by Nocentini et al. [27] provided empirical evidence about the role of contextual (i.e., parental mental health and domestic violence) and relational family processes (i.e., child abuse and neglect, maladaptive parenting, communication, parental involvement and support) in affecting the risk of bullying others or being victimized. However, although the authors found that domestic violence exposure has a more consistent and stable role as a predictor of bullying across studies, to date, the studies specifically focused on the relation between violence exposure within the family and bullying perpetration are still rather scarce.

Based on a review by Voisin and Hong [29], some studies provide empirical evidence that youths who are exposed to domestic violence are more likely to perpetrate school bullying and to become victims of bullying (e.g., [25,60,61,62,63,64,65]). Otherwise, other studies (e.g., [26]) have found an association of domestic violence exposure with other types of problematic behaviors (i.e., externalizing behavior or physical aggression and internalizing behaviors), rather than relational bullying behaviors (e.g., social exclusion, spreading rumors). More specifically, among a large sample of Italian adolescents and preadolescents, Baldry [25] documented that about one-sixth had witnessed domestic violence, and bullies were more likely than not to have witnessed violence in the home, over and above age, gender, and child abuse. Other cross-sectional studies reported similar findings revealing that domestic violence witnessing represents a risk factor for bullying peers [61,62,63,64,65]. Otherwise, in a cross-sectional and retrospective study with undergraduate students from the USA, Sanders and Jenkins [28] found that domestic violence witnessing significantly predicted the frequency of bullying victimization and the presence of relational bullying victimization but not of bullying perpetration.

Conversely, among longitudinal studies, Bowes et al. [60], using a nationally representative community-based sample of British children and distinguishing the differential effects of direct victimization (i.e., child maltreatment) and exposure to domestic violence, found that over and above other socio-environmental factors and children’s behavior problems, youths who were victims of parents’ maltreatment were at increased risk for bullying victimization, whereas those who witnessed domestic violence were at increased risk for bullying perpetration.

Finally, a longitudinal study [26] using a community-based sample of North American children found that witnessing domestic violence was related to several problematic behaviors (i.e., externalizing behavior or physical aggression and internalizing behaviors) but not to child-reported relational bullying behaviors or victimization by peers.

To date, relatively few studies [66,67] have investigated how school bullying is influenced by experiences in environments outside of more immediate or proximal settings (e.g., school and family) and by the perceptions of the neighborhood/community where youths live, such as community violence exposure [12]. In this regard, some studies, without distinguishing the differential effects of community violence witnessing and victimization, found a significant association between violence exposure within the community and bullying perpetration [30,34] over and above other socio-environmental factors, such as poverty, inequality, and political violence [31]. A seminal cross-sectional study by Schwartz and Proctor [33] with a sample of children from an urban area of South Central Los Angeles found that those who had been witnesses to or victims of community violence were more likely to bully their classmates.

Furthermore, using a latent transition mixture analysis, Davis et al. [32] reported that the largest proportion (25%) of North American youths who experienced heightened levels of community violence as witnesses were more likely to be perpetrators of school bullying. Nonetheless, in a study from a medium-sized county in Sweden, Andershed et al. [68] found that bullying others in school was related to a heightened risk of being violently victimized when out on the streets among both boys and girls. In a longitudinal study from Southern Italy [12], it was found that being exposed to community violence as a witness but not as a victim promoted the perpetration of bullying over time.

From a social-ecological systems perspective, and consistent with a transactional developmental model of conduct problems [69], both family and community violence exposure and their effects on child development can be understood as resulting from reciprocal interactions between the individual and environment that are continuously influenced by experiences and conditions across multiple interrelated systems over time [70]. In this regard, there is some empirical support for causal, bidirectional influences between violence exposure and externalizing problems, such that the more youths are exposed to violent contexts, the more likely they learn pro-violence behavioral models, and vice versa; the more youths engage in aggressive and delinquent behaviors, the more likely they are to put themselves in high-risk situations in which they could become witnesses to or victims of violence [20,21].

However, the causal nature of the relationship between exposure to violence, both in the family and neighborhood/community, and bullying remains unclear [46], given the limited number of studies that have examined the mechanisms through which violence exposure could affect involvement in bullying behavior.

### 1.2. Self-Serving Cognitive Distortions as Individual Social-Cognitive Risk Factors for School Bullying Perpetration

Consistent with the social-cognitive approach (e.g., [71,72]), according to which people act upon their interpretation of social events, previous research claimed that moral cognitions represent a key factor for motivating moral or immoral acts such as bullying behaviors [23,73]. In terms of moral cognitive processes, self-serving CDs, defined as “inaccurate or biased ways of attending to or conferring meaning upon experiences” [74] (p. 1), represent one of the common limitations characterizing antisocial youth’s social cognitions [14,75,76].

According to Gibbs and colleagues’ “three *D*s” formulation [77], self-serving CDs are conceived as potentially preceding antisocial action, i.e., primary CDs, as well as following behavior, i.e., secondary CDs. More specifically, though the primary distortions (in the category of self-centered) reflect more immature moral judgment stages [78] and serve as main motivators or “pretexts” of aggressive behaviors, the secondary distortions (in the categories of blaming others, minimizing/mislabeling, and assuming the worst) support self-centered attitudes [14] and have been characterized as pre- or post-transgression rationalizations or “excuses” for facilitating aggressive behaviors.

Despite an increasing number of studies [22,23,24] finding a link between self-serving CDs and externalizing behaviors, only a few studies [12,35] have examined the association between CDs and bullying at school. A study by Owens et al. [35] carried out with Australian adolescents found that bullies and victims of bullies showed a higher tendency than victims and uninvolved persons in assuming the worst, exhibiting minimizing/mislabeling and self-centered CDs, whereas only bullies were higher in blaming others. Similarly, in a study with Italian adolescents, Dragone et al. [12] showed that the development of CDs promoted the perpetration of bullying over time.

However, a transactional developmental model [69] would be better equipped to explain the emergence of chronic antisocial behavior over time. This model suggests the possibility of a multidirectional causality between individual cognitions and behaviors, providing support for Gibbs’ conceptualization of secondary CDs as a form of post-rationalizations or “excuses” serving to emotionally and cognitively overcome dissonance between individual moral standards and behavioral transgressions and neutralize potential feelings of guilt or empathy towards the victim, thus avoiding damage to one’s self-image when engaging in antisocial conducts [79,80].

Based on these considerations, it is necessary to disentangle the temporal order to provide key evidence on the causal direction of the link between moral cognitions and aggressive behaviors [81]. However, the experimental or longitudinal designs suited to test assumptions of temporal order and to clarify a possible causal link between aggression and moral cognitions [82] (p. 45) are still rather scarce. Specifically, in their study with a sample of Italian adolescents, Aquilar et al. [83] found reciprocal influences over time among values, moral judgment—considered similarly to CDs as a moral motivator [84] of externalizing behaviors—and antisocial behaviors. Moreover, with a sample from Switzerland, Ribeaud and Eisner [81] used two-period path models to test the relationship between moral neutralization and aggressive behavior in early adolescence and found that moral neutralization might be envisaged as facilitating aggressive behavior by providing ex ante justifications. In contrast, aggressive behavior would, in turn, induce ex post legitimizations that allow for a smooth integration of norm-breaking behavior into an apparently intact moral self-concept.

### 1.3. Examining How the Cognitive Desensitization Process Could Link Exposure to Violent Contexts, Self-Serving Cognitive Distortions, and School Bullying Perpetration

According to social learning theory [85], children chronically exposed to violence in their daily life environment may learn, via an observational process, that violence itself is a socially acceptable means for conflict resolution [86]. Such a process resulting from growing up in violent contexts and facilitating more approving violent beliefs, more positive moral evaluations of aggressive acts, and more justification for inappropriate behavior inconsistent with social and individual’s moral norms has been defined by Huesmann and Kirwil [37] as “cognitive desensitization to violence.” Consistently, Ng-Mak and colleagues [39] formulated a “pathologic adaptation” model according to which repeated exposure to high levels of violence in inner-city urban neighborhoods leads to cognitions that normalize violence through mechanisms of neutralization of moral standards, which in turn facilitate engagement in future episodes of violence, thus perpetuating the cycle of violence.

The depiction of moral cognitive processes as mediators of life experiences and as proximal mechanisms for externalizing behaviors is consistent with the biopsychosocial perspective on the development of adolescent conduct problems [69]. According to this perspective, it is assumed that as a function of aggressogenic life experiences, such as the repeated experience of being a witness or a victim of violence, children develop idiosyncratic social knowledge about their world and social information processing patterns that justify the appropriateness of behaving aggressively in problematic social situations.

However, to date, relatively few studies have investigated whether the moral cognitive processes could mediate the effect of early life experiences, such as violence exposure within the family and community, on later behavioral problems, such as bullying, also disentangling the potential differential effects due to victimization and witnessing violence (e.g., [33]). Specifically, the transmission of violence among adolescents who directly suffer maltreatment or are indirectly exposed to domestic violence could be cognitively mediated [87]. In this regard, some studies (e.g., [88,89,90]) found that though experiencing witnessing was more likely associated with schemas of justification of violence, which in turn lead to aggressive behavior, direct victimization was linked with less aggressiveness and more depression through the schema of mistrust [89] and with later bullying victimization in the school through the development of maladaptive schemas of rejection [91].

Nevertheless, it was also found that direct victimization experiences within the family, such as childhood maltreatment and physical abuse, might contribute to the development of cognitive structures or schemas in the victims, which in turn would influence their subsequent behavior. Such schemas assume the form of normative beliefs about the social appropriateness of aggression [92], including the idea that the use of the aggression is justified (e.g., because the other deserves it), and lead to positive outcomes for the individual (e.g., because it serves them to obtain respect from others). Furthermore, when exposed to childhood maltreatment and physical abuse, these socio-cognitive biases could also take the form of hostile attributional bias when faced with the ambiguous intentions of others in social situations [93] or of acceptance of violence as normative in adult relationships [94].

Also, with regard to the link between violence exposure within the neighborhood/community and social-cognitive processes, research findings have been mixed. For instance, using qualitative data from male violent offenders from the USA, Wilkinson and Carr [95] found that individuals respond to exposure to violence in many ways, without distinguishing between violence witnessing and victimization. Some of these would be consistent with traditional concepts of moral disengagement. In the same direction were the results of Hyde et al. [43], who found a positive association between neighborhood impoverishment and moral disengagement in an American sample.

Nonetheless, several studies have found significant associations between community violence and the acceptance of violent cognitions or bias of social information processing (see, for example, [40,41]), whereas in their study with Italian adolescents, Bacchini et al. [96] showed that higher levels of exposure to community violence as a witness, along with the perception of higher levels of deviancy among peers, reduced the strength of moral criteria for judging moral violations. Among the very few longitudinal studies from Italy, Esposito et al. [97] showed that a high frequency of exposure to community violence was a significant risk factor for being in the class with higher and tendentially stable CDs, relative to the moderate and decreasing class. Similarly, Dragone et al. [12] found a longitudinal relationship between community violence witnessing and the development of CDs.

Other studies [33] have shown distinct mediational pathways linking each form of violence exposure to social difficulties with peers, suggesting that the impact of victimization on aggressive behavior took place through impairments in emotion regulation, whereas witnessing influences aggressive behavior through social-cognitive biases about aggression when involved in processing social situations. Consistently, Esposito et al. [98] found that a moderate exposure to community violence, as a witness and a victim and as only a witness, were linked to increased bullying perpetration through greater moral disengagement. Interestingly, high levels of exposure as both a witness and a victim did not show significant associations with either moral disengagement or bullying perpetration.

Taking this evidence as a starting point, further longitudinal research is needed to clarify whether both experiences of violence exposure, as a victim and/or as a witness, are associated with constructs of moral cognitions such as CDs, which in turn could promote the involvement in aggressive behaviors such as school bullying perpetration.

### 1.4. The Current Study

The main aim of this study was to investigate the mediating role of self-serving CDs in the relationship between violence exposure within the family and neighborhood/community, as a victim and as a witness, and school bullying perpetration over time. More specifically, we expected to find the following: being exposed to violence within the family and neighborhood/community increases the likelihood that adolescents perpetrate bullying (Hypothesis 1) and develop self-serving CDs (Hypothesis 2); and (ii) making use of CDs promotes engagement in future episodes of bullying perpetration (Hypothesis 3). In addition, consistent with the transactional developmental model, we expected that the associations between both domestic and community violence exposure and CDs and bullying perpetration and those between CDs and bullying perpetration were reciprocal over time (Hypothesis 4).

As regards the specific hypotheses we made about the differential effects of direct victimization and witnessing, for domestic violence exposure, no a priori hypotheses were formulated due to the limited and conflicting prior literature; however, with regard to community violence exposure, we hypothesized significant associations between violence witnessing and both CDs and bullying perpetration, whereas no a priori hypotheses were formulated for violence victimization due to the limited prior literature.

Given that gender-based differences have been observed in violence exposure and its developmental effects [25,99,100] as well as in CDs [35] and bullying behavior [101], adolescent gender was included as a control variable. Adolescents’ social desirability was also used as a control variable, given its potential confounding effect on all study variables [102].

## 2. Materials and Methods

### 2.1. Participants

Participants were drawn from a longitudinal research project (Arzano Longitudinal Project, ALP) that began in 2013 and was aimed at investigating the determinants and pathways of typical and atypical development from early to late adolescence. The study design originally involved sixth and ninth graders of the middle and high schools of Arzano, a relatively small town located in the metropolitan area of Naples. This area is characterized by serious social problems such as high unemployment, high school dropout rates, and the presence of organized crime, with rates that are among the highest in Italy [103].

The sample for the current study consisted at Time 1 (T1) of 778 adolescents (346 males and 432 females; *M_age_* (T1) = 14.20, *SD* = 0.58) from two cohorts of adolescents who were enrolled in the 9th grade (T1 of the study) in 2013 and 2016, with each one longitudinally assessed from 2013 to 2016 and from 2016 to 2019 (4 data points, one-year intervals), respectively. More specifically, 510 adolescents (258 males and 252 females; *M_age_* (T1) = 14.21, *SD* = 0.58) were part of the first cohort, whereas 268 adolescents (88 males and 180 females; *M_age_* (T1) = 14.18, *SD* = 0.58) were part of the second cohort.

Cohort effects were tested by comparing the mean levels of the main variables at the same age. Specifically, a set of Univariate Analyses of Variance (ANOVAs) found no significant differences between the two cohorts for gender, age, social desirability, and all main variables of the study at T1 and T2; conversely, significant differences between the two cohorts were found for domestic violence victimization and self-serving CDs at T3 (Fs_(1, 662)_ = 8.21 and 16.56, *ps* < 0.01 and 0.001, respectively) and at T4 (Fs_(1, 623)_ = 5.33 and 6.88, *ps* < 0.05 and 0.01, respectively), as well as for community violence witnessing at T4 (F_(1, 623)_ = 4.30, *p* < 0.05). However, although statistically significant, Cohen’s d measure of effect size indicated that such cohort differences were very small in size (all *ds* < |0.33|). Accordingly, the data from the two cohorts were combined.

### 2.2. Procedure

Data collection was authorized by the managers of the schools involved in the study and took place during the spring of 2013 and 2016 (T1), 2014 and 2017 (T2), 2015 and 2018 (T3), and 2016 and 2019 (T4). During this period, no noteworthy events occurred in either cohort of adolescents.

Parents’ written consent and adolescents’ assent were obtained prior to the administration of questionnaires. All the administrations were conducted during classroom sessions by trained assistants. Participants were informed about the voluntary nature of participation and their right to discontinue at any point without penalty.

The study was approved by the Ethical Committee of Psychological Research of the Department of Humanities Studies, University of Naples “Federico II”.

The American Psychological Association’s ethical standards regarding research with human subjects were followed throughout the research design and implementation.

### 2.3. Attrition and Missing Data Analysis

In the overall sample, the participation rate was approximately 80% across all time points, with 71 (9.1%), 114 (14.7%), and 153 (19.7%) adolescents from the original sample (N = 778) not assessed at T2, T3, and T4, respectively. Similar participation rates were found for each cohort, as reported in the Supplemental Materials (see Table S1).

The total attrition was mainly due to school dropouts. Although the attrition rate seems very high, it is in line with the latest national statistics on school dropout rates [104]. The Little’s test [105] for data missing completely at random (MCAR) in SPSS 21 (IBM Corp., Armonk, NY, USA) was significant (χ^2^ (90) = 134.19, *p* < 0.01), indicating that data were not missing completely at random. Subsequent *t*-test analyses showed that participants who were missing at T2 and/or T3 and/or T4 significantly reported higher levels of community violence witnessing and CDs at T1; those who were missing at T2 and/or at T3 also significantly reported higher levels of bullying perpetration at T1; and those who were missing at T3 also significantly reported higher levels of domestic violence witnessing and community violence victimization at T1 than participants who had data at all assessments (*ps* ≤ 0.05). Accordingly, Full Information Maximum Likelihood (FIML) was used to handle missing data, enabling us to include all available data in the analyses.

### 2.4. Measures

#### 2.4.1. Exposure to Domestic Violence

At each time point of the study, a reduced version of the adaption for the Italian context by Baldry [25] of the Conflict Tactics Scale [106] was used. Each scale included six items for which participants were asked to indicate, using a 5-point Likert scale (from 1 = Never to 5 = More than five times), the frequency of their being the witness of violence (i.e., verbal or physical) by each one of their parents against the other parent or the target victim of violence (i.e., verbal or physical) by each of their parents during the last year. Sample items were “He or she insulted or said bad words to her or him” and “He or she insulted you or said bad words to you” for domestic violence witnessing and victimization, respectively.

The factor structure of the scale at T1 was tested through Confirmatory Factor Analysis (CFA). The two-factor model, including the correlation between the two factors (i.e., exposure to domestic violence as a witness and as a victim), showed acceptable model fit, χ^2^ (43) = 225.21, *p* < 0.001; CFI = 0.90, RMSEA = 0.07 90% C.I. [0.06, 0.08].

The instrument demonstrated good reliability, with Cronbach’s alphas and McDonald’s Omega coefficients ranging from 0.83 to 0.89 and 0.84 to 0.87, and from 0.86 to 0.91 and 0.85 to 0.91, for violence witnessing and victimization, respectively.

#### 2.4.2. Exposure to Community Violence

At each time point of the study, we used two adapted scales for the local context (exposure to community violence questionnaire; [20]) of the Community Experience Questionnaire by Schwartz and Proctor [33]. Each scale included six items for which participants were asked to indicate, using a 5-point Likert scale (from 1 = Never to 5 = More than five times), the frequency of their being the witness or the victim of violence in the neighborhood. Sample items were “How many times have you seen somebody get robbed?” and “How many times have you been chased by gangs, other kids, or adults?” for violence witnessing and victimization, respectively.

This measure has been previously administered to Italian adolescents [20,98] and has shown good reliability and solid psychometric properties. The factor structure of the scale at T1 was tested through a CFA. The two-factor model, including the correlation between the two factors (i.e., exposure to community violence as a witness and as a victim), showed an acceptable fit when allowing four error covariances in the model, χ^2^ (49) = 207.88, *p* < 0.001; CFI = 0.92, RMSEA = 0.06 90% C.I. [0.05, 0.07].

The instrument demonstrated good reliability, with Cronbach’s alphas and McDonald’s Omega coefficients ranging from 0.81 to 0.92 and 0.79 to 0.92, and from 0.79 to 0.92 and 0.82 to 0.92, for violence witnessing and victimization, respectively.

#### 2.4.3. Self-Serving Cognitive Distortions (CDs)

At each time point of the study, participants were asked to respond to the 39 items of the How I think Questionnaire (HIT; [74]; Italian validation by [107]), measuring self-serving CDs. For each item, participants were asked to indicate their agreement on a 6-point Likert scale (from 1 = Disagree strongly to 6 = Agree strongly). Sample items were “People need to be roughed up once in a while” and “Everybody breaks the law, it’s no big deal”.

A composite score was obtained by averaging item ratings, with a higher score reflecting higher levels of CDs.

Previous studies corroborated the validity of this measure with adolescent samples in the Italian context [97,107].

The CFA performed at T1 with items grouped in four parcels (assessing the four categories of CDs) showed acceptable model fit, χ^2^ (2) = 7.05, *p* < 0.05; CFI = 0.99, RMSEA = 0.06 90% C.I. [0.02, 0.11].

Cronbach’s alphas and McDonald’s Omega coefficients ranged from 0.95 to 0.97 across all time points, respectively, thus indicating good reliability of the scale.

#### 2.4.4. School Bullying Perpetration

At each time point of the study, bullying perpetration was self-reported by adapting the classical Florence Bullying and Victimization Scales (FBVSs; [108]). For the purposes of the present study, we only used data about bullying. Adolescents were provided with a definition of bullying as intentional, repetitive aggressive behaviors including some sort of power imbalance between those involved and were asked to indicate, using a 5-point Likert scale (from 1 = Never to 5 = Several times a week), the frequency with which, since the beginning of the school year, they had exhibited eight different bullying behaviors, direct (i.e., physical, e.g., hitting/kicking, “I hit, kicked, or punched someone” and verbal, e.g., threatening, “I threatened someone”) and indirect (e.g., excluding/ignoring, “I made nicknames for others that they didn’t like”).

A composite score was obtained by averaging item ratings, with a higher score reflecting higher involvement in bullying behaviors as a perpetrator.

This scale has been administered to Italian adolescents in prior studies (e.g., [98]), showing good reliability and solid psychometric properties.

The CFA at T1 showed an acceptable fit when allowing one error covariance in the model, χ^2^ (19) = 55.52, *p* < 0.001, CFI = 0.93, RMSEA = 0.05 90% C.I. [0.04, 0.07].

Cronbach’s alphas and McDonald’s Omega coefficients ranged from 0.84 to 0.89 across all time points, respectively, thus indicating good reliability of the scale.

#### 2.4.5. Control Variables

Information about the sociodemographic characteristics of the sample were obtained by asking participants to indicate their own age and gender (1 = male, 2 = female). At T1 of the study, participants were also asked to complete 12 items from the Lie scale of the Big Five Questionnaire [109]. Each item was rated on a 5-point Likert scale (from 1 = Very false for me to 5 = Very true for me). Sample items were “I’ve always gotten along with everyone” and “I’ve never told a lie”.

A composite score was obtained by averaging item ratings, with higher scores reflecting higher levels of socially desirable responding. Cronbach’s alphas and McDonald’s Omega coefficients were 0.97 at T1, respectively.

### 2.5. Analytic Strategy

**Figure 1:**
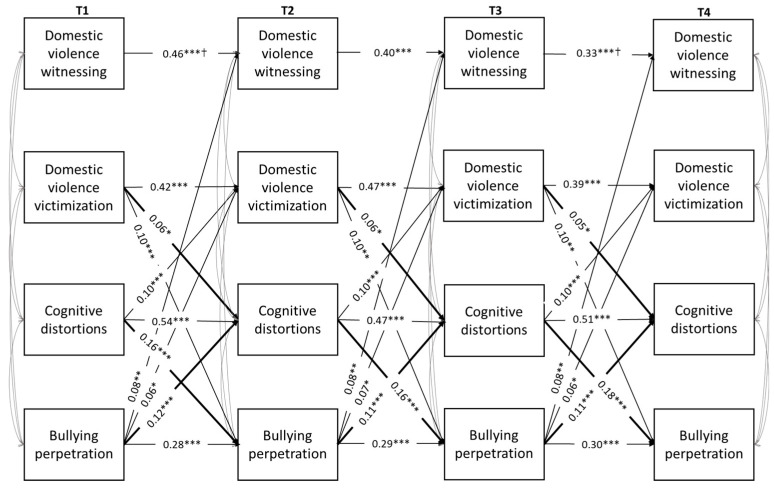
Cross-lagged mediational model with domestic violence exposure as a witness and as a victim. Reported coefficients refer to standardized estimates. Bold lines represent significant indirect paths. For the sake of simplicity, nonsignificant paths and relations with control variables are omitted. †, represents statistically significant differences in paths across time. * *p* < 0.05, ** *p* < 0.01, *** *p* < 0.001.

**Figure 2:**
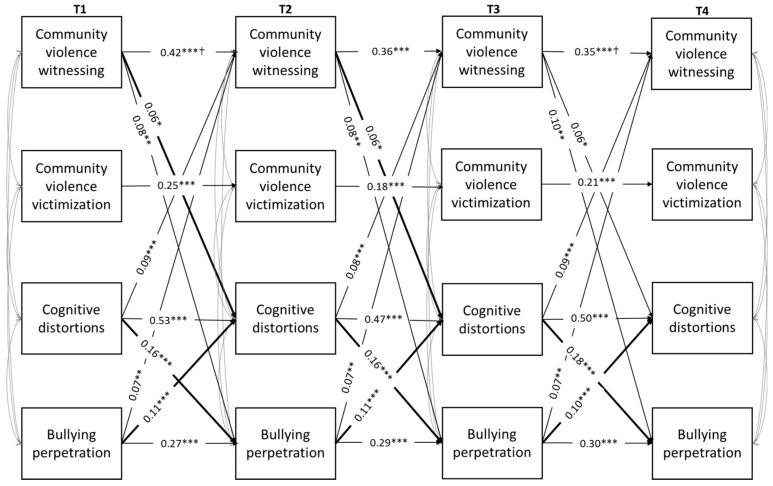
Cross-lagged mediational model with community violence exposure as a witness and as a victim. Reported coefficients refer to standardized estimates. Bold lines represent significant indirect paths. For the sake of simplicity, nonsignificant paths and relations with control variables are omitted. †, represents statistically significant differences in paths across time. * *p* < 0.05, ** *p* < 0.01, *** *p* < 0.001.

Preliminarily, concurrent and longitudinal associations among study variables were performed through Pearson correlations. Subsequently, four-wave cross-lagged panel analyses were used to test the hypothesized longitudinal relations among the study variables (see Figure 1 and Figure 2). Extensive overviews of the use of this model for mediation analyses are given by Cole and Maxwell [110] and MacKinnon [111], as it allows us to better investigate the likely direction of causal influence among variables, test for alternative models, and lessen biases in testing mediation. Specifically, the cross-lagged panel mediation design allowed us to control for baseline values of all variables in each wave and to examine the transactional nature and likely causal direction of the pathways linking exposure to domestic and community violence, CDs, and school bullying perpetration. The analyses were modeled in Mplus 8 [112] using the Maximum Likelihood Estimation with Robust Estimators (MLR), due to the non-normality of domestic violence witnessing, community violence victimization, and bullying perpetration measures (skewness and kurtosis values ranged from 3.25 to 12.03, 3.32 to 12.21, and 3.13 to 11.77, respectively). Missing data were handled by using Full Information Maximum Likelihood (FIML) estimation of the parameters. As indicated in a previous work [113], FIML is an especially useful missing data treatment in longitudinal designs, because the outcome scores for dropouts tend to be correlated with their own previously recorded responses from earlier waves (i.e., an MAR pattern).

Two models were run separately for each specific daily life context: one for exposure to violence within the family and the other for exposure to violence within the neighborhood/community, both as a witness and as a victim. The models included correlations among concurrent constructs at all time points, autoregressive paths for each construct across time, and all cross-lagged paths. Adolescent gender and social desirability were included in the models as observed covariates to ensure that the associations between the variables were adjusted for their potential confounding effects.

Several indexes were used to evaluate the goodness of fit: the Yuan–Bentler [114] scaled chi-square statistic (YBχ^2^), the Comparative Fit Index (CFI; [115]), the Tucker–Lewis Index (TLI; [116]), and the Root Mean Square Error of Approximation, with associated 90% confidence intervals (RMSEA; [117]). A CFI ≥ 0.90 and RMSEA ≤ 0.08 indicate a model’s acceptable fit to the data [118]. To test equivalence of the structural parameters across time, two nested models were considered: a baseline model, in which parameters were freely estimated across time, and a fully constrained model, in which the structural paths and correlations among concurrent constructs were constrained to be equal over time. The Satorra–Bentler chi-square difference test (ΔSBχ^2^) was used to test relative fit of nested models [119]. When the more constrained model was rejected, a less restrictive model of partial invariance was tested in which, in accordance with modification indices, equality constraints on one or more parameters were relaxed until the change in fit was no longer significant.

## 3. Results

### 3.1. Correlations among Study Variables

Means, standard deviations, and Pearson correlations among all study variables are shown in Table 1a,b. For the sake of clarity, we reported correlations separately for each specific daily life context: one for exposure to violence within the family (see Table 1a), the other for exposure to violence within the neighborhood/community (see Table 1b). As can be seen in Table 1a,b, stability across adjacent time points was moderate for all study variables, with some variability in correlation coefficients that were higher for CDs (r ranging from 0.53 and 0.62), domestic violence victimization (r ranging from 0.41 to 0.58), and community violence witnessing (r ranging from 0.36 and 0.45). Furthermore, all study variables were significantly and concurrently intercorrelated with each other at all time points. Specifically, we found that both domestic and community violence exposure (as a witness and as a victim), respectively, were positively associated with both CDs and bullying perpetration, which were associated with each other within the same time (all *ps* < 0.001). Significant associations were also found among the study’s variables across time, although with a slightly lower magnitude.

### 3.2. Cross-Lagged Panel Modeling

#### 3.2.1. Exposure to Domestic Violence as a Witness and as a Victim

The model with all autoregressive and cross-lagged paths, as well as correlations among concurrent constructs freely estimated across time, showed an adequate fit to the data, YBχ^2^(48) = 238.74, *p* < 0.001; CFI = 0.94; RMSEA = 0.07, 90% C.I. [0.06, 0.08].

Imposing equality constraints to autoregressive and cross-lagged paths and correlations among concurrent constructs in order to test their invariance over time led to a significantly worse model fit, ΔSBχ^2^(74) = 141.46, *p* < 0.001. In accordance with modification indices, the equality constraints on the autoregressive paths between domestic violence witnessing at T1 and T2 and at T3 and T4 on the cross-lagged paths linking T1 social desirability with T1 and T4 CDs and on the correlations between domestic violence witnessing and victimization at T3 and T4 were relaxed in order to improve the model fit, as were those between domestic violence witnessing and victimization with bullying perpetration at T3 and T1, respectively, those between domestic violence victimization and CDs at T4, and finally, those between CDs and bullying perpetration at T1 and T2, YBχ^2^(111) = 327.10, *p* < 0.001; CFI = 0.93; RMSEA = 0.05, 90% C.I. [0.04, 0.06]. Thus, the partially constrained model did not differ significantly from the freely estimated model, ΔSBχ^2^(63) = 76.64, *p* = 0.12. Significant paths and standardized coefficients for the final model are shown in Figure 1.

As can be noted in Figure 1, all variables were correlated with each other within at all time points. As regards the stability of study variables across time, all measures showed moderate to high stability over time, with the highest levels of stability emerging for CDs and domestic violence victimization. Regarding cross-lagged paths, significant associations between domestic violence victimization and both CDs and bullying perpetration at each time point were found; conversely, bullying perpetration significantly predicted domestic violence witnessing, but not vice versa. Moreover, CDs significantly predicted bullying perpetration at each time point. Bidirectional relations between domestic violence victimization and both CDs and bullying perpetration and between CDs and bullying perpetration over time were also found.

Finally, as can be seen in Table 2, the mediation analyses highlighted marginally significant indirect effects from T1 and T2 domestic violence victimization to T3 and T4 bullying perpetration through T2 and T3 CDs, respectively (β = 0.01, *p* < 0.05, 95% C.I.s [0.002, 0.017] and [0.002, 0.018], respectively). Moreover, the mediation analyses confirmed a series of reciprocal associations between CDs and bullying perpetration over time, such that earlier high CDs at T1 and T2 increased CDs at T3 and T4 through the mediation of bullying perpetration at T2 and T3, respectively (β = 0.02, *p* < 0.001, 95% C.I. [0.01, 0.03], respectively). Further, an earlier involvement in bullying at T1 and T2 increased the tendency to bully others at T3 and T4 through the mediation of CDs at T2 and T3, respectively (β = 0.02, *p* < 0.001, 95% C.I. [0.01, 0.03], respectively).

#### 3.2.2. Exposure to Neighborhood/Community Violence as a Witness and as a Victim

The model with all autoregressive and cross-lagged paths, as well as correlations among concurrent constructs freely estimated across time, showed an adequate fit to the data, YBχ^2^(48) = 143.33, *p* < 0.001; CFI = 0.96; RMSEA = 0.05, 90% C.I. [0.04, 0.06].

Imposing equality constraints to autoregressive and cross-lagged paths and correlations among concurrent constructs in order to test their invariance over time led to a significantly worse of the model fit, ΔSBχ^2^(74) = 182.02, *p* < 0.001. In accordance with modification indices, the equality constraints on the autoregressive paths between community violence witnessing at T1 and T2 and at T3 and T4 on the cross-lagged paths linking T1 social desirability with T1 and T4 CDs as well as gender with T4 community violence witnessing were relaxed in order to improve the model fit, as were the correlations between community violence witnessing and victimization at each time point, those between community violence witnessing and CDs at T1 and T3 as well as with bullying perpetration at T1, and those between CDs and bullying perpetration at T2, YBχ^2^(110) = 225.91, *p* < 0.001; CFI = 0.95; RMSEA = 0.04, 90% C.I. [0.03, 0.04]. Thus, the partially constrained model did not differ significantly from the freely estimated model, ΔSBχ^2^(62) = 79.10, *p* = 0.07. Significant paths and standardized coefficients for the final model are shown in Figure 2.

As can be noted in Figure 2, all variables were correlated with each other at all time points. As regards the stability of study variables across time, all measures showed moderate to high stability over time, with the highest levels of stability emerging for CDs and community violence witnessing. Regarding cross-lagged paths, significant associations between community violence witnessing and both CDs and bullying perpetration at each time point were found, and CDs significantly predicted bullying perpetration at each time point. Moreover, a series of bidirectional relations between community violence witnessing and both CDs and bullying perpetration as well as between CDs and bullying perpetration over time were also found.

Finally, as can be seen in Table 2, the mediation analyses highlighted marginally significant indirect effects from T1 and T2 community violence witnessing to T3 and T4 bullying perpetration through T2 and T3 CDs, respectively (β = 0.01, *p* < 0.05, 95% C.I.s [0.001, 0.018] and [0.001, 0.020], respectively). Moreover, the mediation analyses confirmed a series of reciprocal associations between CDs and bullying perpetration over time, such that earlier high CDs at T1 and T2 increased CDs at T3 and T4 through the mediation of bullying perpetration at T2 and T3, respectively (β = 0.02, *p* < 0.001, 95% C.I.s [0.01, 0.03] and [0.01, 0.02], respectively). Further, an earlier involvement in bullying at T1 and T2 increased the tendency to bully others at T3 and T4 through the mediation of CDs at T2 and T3, respectively (β = 0.02, *p* < 0.001, 95% C.I. [0.01, 0.03], respectively).

#### 3.2.3. Control Variables

With respect to covariates, adolescent gender was negatively related to community violence, both as a witness and as a victim, at T1 (βs = −0.07 and −0.05, *ps* < 0.001 and < 0.05, respectively), T2 (βs = −0.07 and −0.05, *ps* < 0.001 and < 0.05, respectively), and T3 (βs = −0.06 and −0.04, *ps* < 0.001 and < 0.05, respectively), and to community violence victimization also at T4 (β = −0.04, *p* < 0.05), as well as to CDs and bullying perpetration at each time point, at T1 (βs = −0.10 and −0.17, *p* < 0.001, respectively), T2 (βs = −0.10 and −0.16, *p* < 0.001, respectively), T3 (βs = −0.09 and −0.16, *p* < 0.001, respectively), and T4 (βs = −0.09 and −0.16, *p* < 0.001, respectively), with males scoring higher than females.

As regards the social desirability bias, negative associations were found with violence victimization both in the family and in the neighborhood/community at each time point, at T1 (βs = −0.08 and −0.06, *ps* < 0.001 and < 0.05, respectively), T2 (βs = −0.07 and −0.06, *ps* < 0.001 and < 0.05, respectively), T3 (βs = −0.08 and −0.05, *ps* < 0.001 and < 0.05, respectively), and T4 (βs = −0.07 and −0.04, *ps* < 0.001 and < 0.05, respectively), as well as with CDs at T1 (β = −0.20, *p* < 0.001) and bullying perpetration at each time point (all βs = −0.07, *ps* < 0.001), with youths more careful about their social image scoring lower on such constructs.

## 4. Discussion

Guided by the social-ecological model [9], we used a four-wave cross-lagged panel design to investigate the longitudinal and simultaneous pathways linking violence exposure within the family and neighborhood/community, through witnessing and direct victimization and self-serving CDs, with school bullying perpetration during adolescence. Furthermore, we tested the cognitive desensitization process that could develop in response to chronically violent contexts [36,37,38,39] by considering moral CDs as indicative of cognitive desensitization that would occur after repeated experiences of domestic and community violence exposure. Since the transactional developmental model is best equipped to describe the emergence of chronic antisocial behavior across time [69], we also examined the reciprocal associations between both domestic and community violence exposure with CDs and bullying perpetration, as well as between CDs and bullying perpetration over time. All the effects were examined controlling for adolescent gender and social desirability bias.

Taking into account the co-occurrence of multiple contexts and experiences through which youths are exposed to violence [50], a specific contribution of our study concerned the detection of differential effects of multiple contexts (i.e., the family and the neighborhood/community) and multiple forms (i.e., through witnessing and direct victimization) of violence exposure linked to self-serving CDs and bullying behaviors in adolescence.

Our findings showed that exposure to violence both within the family and community were associated with self-serving CDs and bullying perpetration over time, although through different forms; more specifically, for domestic violence exposure, this occurred through direct victimization (i.e., child maltreatment or abuse), whereas for community violence exposure, it occurred through witnessing. Moreover, we found a significant association between self-serving CDs and bullying perpetration over time. Each of these longitudinal patterns was found to have a bidirectional direction.

Finally, as regards the cognitive desensitization process we tested, a significant mediating role of self-serving CDs in the relationship between domestic violence victimization and community violence witnessing was found, respectively, with school bullying perpetration.

Taken together, these findings provide a relevant contribution to the prior literature on the environmental precursors of pro-violence moral cognitions. This offers some practical insights for counteracting externalizing behaviors in adolescence, such as bullying, which has been widely recognized as a serious public health concern.

### 4.1. Social-Environmental Risk Factors for School Bullying Perpetration: The Role of Domestic and Community Violence Exposure

Consistent with the concept of equifinality and in line with the Hypothesis 1, we found that both domestic and community violence exposure were associated with bullying perpetration over time, confirming a basic postulate that “violence breeds violence” [13]. However, we found differential effects of multiple forms (i.e., through witnessing and direct victimization) of violence exposure to bullying perpetration in adolescence. More specifically, as regards domestic violence exposure, our findings that direct victimization rather than witnessing was associated with bullying perpetration are consistent with a previous cross-sectional study by Bacchini et al. [15] highlighting that being a victim of domestic violence had a stronger concurrent association with antisocial behavior than witnessing violence at home and, partially, in accordance with another study by Holt et al. [63], who found higher rates of child maltreatment for both bullies and victims.

Similarly to our findings, the review by Nocentini et al. [27] found that experiencing child abuse and maltreatment by parents was the most consistent family risk variable to be associated with bullying perpetration.

Conversely, other cross-sectional [25,61,62,64,65] and longitudinal [60] studies emphasized a stronger impact of domestic violence witnessing in promoting bullying perpetration, whereas Bauer et al. [26] found, in their longitudinal study, that although witnessing domestic violence was related to increased problematic behaviors (i.e., externalizing behavior or physical aggression and internalizing behaviors), it was associated neither with child-reported bullying behaviors nor with victimization by peers.

The lack of effects of witnessing domestic violence on bullying perpetration could be explained by referring to the changes that occur in family influence during adolescence, characterized by a significant increase in other socialization agents, such as peers [120], and by more time spent in environments outside the home, such as the neighborhood/community. For this reason, as Mrug and Windle [52] argued, the higher probability of being a witness to community violence, especially as young people grow, may desensitize them to the effects of violence they witness at home, putting them at lower risk of development externalizing behaviors (e.g., delinquency; [58]) such as bullying.

Also, it should be noted that the model we tested considered the concurrent effects of both witnessing and victimization in the domestic setting. The observed correlations suggested that there could be an overlap between experiences of victimization and witnessing domestic violence. When both variables are considered simultaneously, only victimization shows a significant effect on bullying perpetration. This means that the effect of victimization on bullying behavior is independent of the experience of witnessing violence. On the other hand, the effect of witnessing violence does not hold independently of victimization. In other words, if an individual is a victim of domestic violence, he or she is likely to engage in bullying behavior regardless of whether he or she also witnessed domestic violence. Conversely, merely witnessing domestic violence, without experiencing direct victimization, does not lead to increased bullying perpetration.

However, it is important to note that witnessing domestic violence, such as frequent parental conflicts, may often coincide with being victimized at home [50]. However, a child can be victimized, for example, through harsh parental discipline, without necessarily witnessing domestic violence. Further research could explore the different profiles of exposure to domestic violence and the potential co-occurrence of multiple forms, as well as their effects on bullying perpetration.

Moving on to the effects of community violence exposure, consistent with our expectations and with the previous literature (e.g., [46,66,121,122]) showing that youths who live in neighborhoods judged to be less safe (i.e., characterized by more violent behaviors, where access to guns and gang membership may be more likely) were more likely to engage in externalizing behaviors, we found that experiencing community violence witnessing predicted bullying perpetration over time. This finding is also in line with previous studies specifically focused on bullying behaviors [12,32,33], and as Bowes et al. [60] argued, it could be interpreted by referring to the thesis according to which hostile interactions observed in local communities may provide youths with models of aggressive behaviors that they can reproduce in the context of peer relationships.

Conversely, although the correlations suggested a co-occurrence of violence witnessing and victimization within the community, the lack of victimization effects on bullying perpetration we found may be explained by taking into account those studies that highlighted the fact that being a victim of violence within the community could be linked with other variables that we did not include in the current study, such as impairments in emotional self-regulation [33] and internalizing [58] rather than externalizing symptoms. A recent study by Esposito et al. [98] analyzing profiles of community violence exposure found that community violence victimization only occurred together with witnessing at average and high levels. When examining the association with bullying perpetration, only average exposure—and not high exposure—as both a victim and a witness had a significant direct effect on bullying perpetration compared to the profile with low exposure, thus providing further support for the hypothesis that the impact of exposure to community violence on youths’ developmental outcomes can vary when considering the chronicity of violence exposure.

In addition, perpetrating bullying significantly predicted both domestic and community violence exposure over time. These results support Hypothesis 4, confirming the key assumption of the transactional approach to the development of conduct problems in adolescence [69], according to which it is plausible that a vicious circle takes place between violent contexts and youths’ behavior, so that youths who engage in aggressive behavior are more likely to put themselves in high-risk situations in which they are more likely to be witnesses to violence in the community [20,21] and to be victims of violence by their parents; the latter could lead to recourse to aggressive behavior to discipline their children who bully peers, thus perpetuating the cycle of violence [28]. Indeed, as suggested by Sanders and Jenkins [28], the beliefs and behaviors modeled by parents through experiences of domestic violence are then internalized and accepted by their children, who then mimic these actions in their interactions with peers outside the home.

### 4.2. Pathways Linking Exposure to Domestic and Community Violence, Self-Serving Cognitive Distortions, and Bullying Perpetration: The Cognitive Desensitization Process

In line with social learning theory [85] and its crime-related extension [69], our findings related to the mediation analyses seem to corroborate the cognitive desensitization hypothesis [37], revealing significant indirect effects from domestic and community violence exposure, as a victim and as a witness, respectively, to bullying perpetration through CDs over time. More in detail, we found that experiencing domestic violence victimization and community violence witnessing were associated with the individual tendency to create self-serving CDs (Hypothesis 2), which in turn promoted bullying perpetration over time (Hypothesis 3).

Regarding domestic violence victimization, our findings suggested that intergenerational transmission of violence in adolescents who directly suffer maltreatment by their parents could be cognitively mediated [87], making youth more likely to perceive threats and attribute hostile intent to others rather than benign interpretations, especially when faced with the ambiguous intentions of others in social situations [93]. Therefore, when chronically exposed to parental victimization, youths may develop cognitive schemas that could reflect a tendency towards hypervigilance to perceived threatening cues and a hostile attributional bias that emerges as an adaptive response to actual threats in the past [69,93]. They could also adopt normative beliefs about the social appropriateness of aggression [92].

However, contrary to our results, some empirical evidence (e.g., [88,89]) suggested a more relevant role of domestic violence witnessing in the promotion of the development of justification schemas about violence, whereas direct victimization was linked to the schema of mistrust [89] or rejection as a result of family abuse and victimization [91].

Conversely, when examining the link between violence exposure within the neighborhood/community and social-cognitive processes, our results revealed an association between violence exposure as a witness, but not as a victim, and the individual tendency to create self-serving CDs over time, thus corroborating theoretical assumptions of social learning theory [85] and biopsychosocial perspectives [69]. According to these, youths who are exposed to violence within their living environments learn and internalize via observational learning a series of criminal/deviant models that take the form of social-cognitive schemas, beliefs, and positive attitudes towards violence [36,38].

These findings are consistent with a process of cognitive desensitization to violence [37] and are in line with Anderson’s [123] “Code of the Street” perspective, which suggested that living in neighborhoods where macrostructural patterns of disadvantage are radicalized facilitates access to the street’s subculture, which shapes pro-violence values and beliefs such as CDs to legitimatize the use of violence conceived as an acceptable problem-solving tool in neighborhoods where the street culture is widespread. Furthermore, our findings are consistent with the few studies [12,97] framed within Gibbs and colleagues’ [75] theoretical formulation of CDs but expand on these by exploring the environmental precursors of moral cognitions, considering both more proximal (i.e., the family) and distal (i.e., the community) microsystems of social ecology.

The lack of associations between community violence victimization and self-serving CDs over time is consistent with the findings by Schwartz and Proctor [33], which showed that only violence witnessing was associated with social-cognitive biases supporting positive evaluation of violent behavior, whereas experiencing violence victimization was more likely be associated with impairments in emotion regulation.

Taken together, the findings discussed above provide further support for a “pathologic adaptation” model [39], according to which chronic violence exposure leads to a normalization of violence through the neutralization of moral standards. Therefore, it allows us to speculate that growing up in violent contexts, both in the family and neighborhood/community, might undermine the normative process of moral development, thus causing a moral delay, which consolidates into self-serving CDs [124].

Moreover, in line with Hypothesis 4, our findings also highlighted bidirectional relations between exposure to violence, as a victim and as a witness, within the family and community, respectively, and the development of self-serving CDs, such that the more youths are exposed to violence within the family and community, the more she or he develops biased cognitive processes to justify her or his immoral actions, which in turn puts the individual in high-risk situations in which they are more likely to be victims of parental violence or witnesses of community violence.

These results, along with those above-mentioned about the reciprocal effects of domestic and community violence exposure with bullying perpetration over time, seem to provide support for the lifestyle exposure theory [125], indicating that exposure to violence is not random but is closely related to the behavior (or lifestyle and attitudes) of individuals and to the transactional approach to the development of conduct problems in adolescence [69], according to which it is plausible to hypothesize that a vicious circle takes place between adolescents’ attitudes and behaviors and the risk of being victims and/or witnesses of violence in their daily life environments.

Finally, as regards Hypothesis 3, consistent with our expectations and with the social-cognitive approach (e.g., [71,72]) that links behavior to the way one thinks about situations, we found that the tendency to create self-serving CDs when interpreting social situations promoted engagement in bullying perpetration over time, and vice versa. In line with previous studies (e.g., [126]) developed within the theoretical framework of moral disengagement [71,72], our findings suggest that youths need to construct attitudes and beliefs that justify their immoral actions in order to maintain a positive self-concept. As expected (Hypothesis 4), we also found a predictive role of bullying perpetration on CDs and a recursive association between cognition and behavior over time, such that cognitions affect behavior, and behavior feeds back into cognitions. This result is framed within the transactional developmental model [69] and suggests the possibility of a multidirectional causality between individual cognitions and behaviors, providing support to Gibbs’ conceptualization of secondary CDs as a form of post-rationalization or “excuses” serving to emotionally and cognitively overcome dissonance between individual moral standards and behavioral transgressions. Similarly, Aquilar et al. [83] found a reciprocal influence over time among values, moral judgment (considered similar to CDs as a moral motivator [84] of externalizing behaviors), and antisocial behaviors. Furthermore, our findings are consistent with those of Ribeaud and Eisner [81], who suggested that moral neutralization and aggression could be conceived as the cognitive and as the behavioral expression of the same phenomenon, respectively. Specifically, in the process of (aggressive) decision making, moral neutralization might be envisaged as facilitating aggressive behavior by providing ex ante justifications, whereas aggressive behavior would in turn induce ex post legitimizations that allow for a smooth integration of norm-breaking behavior into an apparently intact moral self-concept.

In addition, the mediation analyses also confirmed a series of reciprocal associations between CDs and bullying perpetration over time, such that the later increase in bullying perpetration was mediated by earlier higher CDs, and vice versa. That is, the later increase in making use of CDs was mediated by earlier higher involvement in bullying perpetration, such that the more a youth makes use of CDs, the more she or he is inclined to perpetrate bullying and, vice versa; the more a youth is involved in bullying perpetration, the more she or he is more likely to increase beliefs in the normative nature of violence [56].

Taken together, our findings, in line with a transactional developmental model [69], corroborate the view of a multidirectional causality between life experiences and individual cognitions and behaviors, such that these constructs reinforce each other over time in the process of (aggressive) decision making [81].

### 4.3. Limitations and Future Directions

In interpreting these findings, several limitations need to be acknowledged. First, the evaluation of all constructs in the study relied exclusively on adolescent self-reporting, which may be subject to social desirability. Despite all the effects in the current study being controlled for social desirability bias, it is known (e.g., [102]) that adolescents are more careful about their social image than other age groups, and they may be unlikely to report behavior that displays them in a negative light. Furthermore, more objective and comprehensive measures of violence in the everyday lives of adolescents, including official data from national census agencies and police departments, may provide a more complete assessment of violence exposure [127]. Future studies may benefit from utilizing a multi-informant approach (e.g., peers’ and teachers’ reports for behavioral constructs) jointly with self-report measures.

Another limitation concerns the generalizability of the results, as the study included a sample from a limited geographical area in Southern Italy characterized by serious social problems and high rates of violence exposure. Such experiences might shape culture-specific beliefs and values that, in turn, might influence an individual’s cognitions and behaviors [15]. For this reason, more research is needed to confirm that the explanatory model proposed in this study applies to populations from other, possibly differing, cultural contexts. In addition, data from our study were collected before the outbreak of the COVID-19 pandemic, which requires us to be more cautious in generalizing our findings at different times. In particular, it is important to recognize that due to the stay-at-home measures following the COVID-19 pandemic (i.e., social isolation, school closures, increased parental control, and reduced access to healthcare), street violence declined [128]; however, the incidents of domestic violence increased [129], with harmful effects on youths’ mental health [130]. Therefore, considering the special circumstances derived from the COVID-19 pandemic, research linking domestic and community violence exposure with behavioral problems in the context of a health crisis is highly recommended.

Furthermore, given the co-occurrence of different kinds of violence exposure from multiple contexts [50] and the moderating role of gender in the relation between violence exposure and psychological outcomes (e.g., [25,99,100]), future research could also investigate the cumulative and interactive effects of domestic and community violence exposure as well of adolescent gender in exacerbating adjustment problems (e.g., [38]). In addition, the lack of witnessing and victimization effects of domestic and community violence, respectively, on self-serving CDs and bullying perpetration could suggest the need to consider other relevant variables, such as impairments in emotional self-regulation (e.g., [33]) and internalizing problems (e.g., [58]). Also, some other school- or classroom-level variables (e.g., peer/teacher support, peer pressure, school climate, etc.) could be relevant to improving our understanding of the dynamics involved in bullying episodes, considering that bullying is a complex social peer group process [131].

Finally, because self-serving CDs might be perceived as relatively stable cognitive mechanisms [75] that could differ among individuals over time, further analytical strategies could be implemented in future studies, such as random intercept cross-lagged panel models that allow for parsing out the between-person stability over time, such that the lagged coefficients represent within-person patterns of change [132].

## 5. Conclusions and Practical Implications

Notwithstanding these limitations, this study provides further corroboration, consistent with Bronfenbrenner’s [9] social-ecological framework, of the joint and reciprocal role of individual and contextual factors implicated in the enactment of school bullying behaviors. More specifically, the findings of the present study highlight the fact that youths who experience violence in their daily life contexts develop pro-aggressive moral cognitions taking the form of self-serving CDs, which in turn amplify the risk for involvement in bullying perpetration.

Overall, these results have a direct impact on health promotion in adolescence, pointing out the need to consider the development of conduct problems (e.g., bullying) in adolescence as a process involving multiple levels of individual ecology and providing useful suggestions for designing and implementing appropriate interventions aimed at preventing and reducing adolescents’ involvement in bullying perpetration by reducing their biased moral cognitions, especially when exposed to violent environments.

In this regard, prevention efforts should pay particular attention to highly violent contexts to which youths are at risk of being exposed during adolescence to maximize the benefits of programs aimed at promoting their well-being and preventing juvenile disease. For instance, interventions designed to reduce violence at home and in the community could help to prevent behavioral problems such as bullying among low-income adolescents.

As suggested by Bordin et al. [100], one possible governmental strategy to reduce exposure to violence at home would be the development of preventive programs in local primary care health units aiming to promote healthy relationships between adolescents and their parents. Similarly, to reduce the time adolescents spend on the streets and the opportunities to become involved with violence and crime, such programs would increase the number of daily hours of public schools, particularly in disadvantaged neighborhoods.

Furthermore, drawing inspiration from our findings related to the process of cognitive desensitization to violence detected among adolescents who suffered violence by their parents or witnessed community violence, educational and healthcare professionals should take an ecological approach to violence to optimize intervention effects and counteract the cognitive mechanisms underlying the cycle of transmission of violence.

An example of a school-based program developed within Gibbs’ theoretical framework and aimed at reducing adolescents’ “thinking errors” or CDs is the “Equipping Youth to Help One Another (EQUIP) for Educators” program (EfE; [133]), whose effectiveness has been recently demonstrated in the Italian context [134]. In accordance with our findings highlighting the key role of CDs in promoting bullying perpetration in youths exposed to violence, Dragone and colleagues [134] provided empirical support for the effectiveness of such psychoeducational programs in promoting a more responsible way of (inter)acting with peers in high-risk school contexts by equipping youths with skills for correcting their “thinking errors” or self-serving CDs when interpreting social events.

## Figures and Tables

**Table 1:** (**a**). Correlations among the study’s variables with exposure to violence within the family. (**b**). Correlations among the study’s variables with exposure to violence within the neighborhood/community.

**(a)**																
	**1**	**2**	**3**	**4**	**5**	**6**	**7**	**8**	**9**	**10**	**11**	**12**	**13**	**14**	**15**	**16**
1. T1 DVW	1															
2. T1 DVV	0.61 ***	1														
3. T1 CDs	0.18 ***	0.26 ***	1													
4. T1 BP	0.18 ***	0.28 ***	0.34 ***	1												
5. T2 DVW	0.54 ***	0.36 ***	0.17 ***	0.24 ***	1											
6. T2 DVV	0.34 ***	0.51 ***	0.21 ***	0.24 ***	0.67 ***	1										
7. T2 CDs	0.08 *	0.19 ***	0.62 ***	0.32 ***	0.24 ***	0.32 ***	1									
8. T2 BP	0.04	0.13 ***	0.23 ***	0.37 ***	0.20 ***	0.23 ***	0.33 ***	1								
9. T3 DVW	0.47 ***	0.33 ***	0.14 ***	0.16 ***	0.48 ***	0.39 ***	0.17 ***	0.11 **	1							
10. T3 DVV	0.32 ***	0.45 ***	0.19 ***	0.20 ***	0.40 ***	0.58 ***	0.30 ***	0.21 ***	0.66 ***	1						
11. T3 CDs	0.06	0.17 ***	0.43 ***	0.23 ***	0.14 ***	0.24 ***	0.53 ***	0.30 ***	0.19 ***	0.31 ***	1					
12. T3 BP	0.11 **	0.23 ***	0.24 ***	0.29 ***	0.16 ***	0.29 ***	0.34 ***	0.42 ***	0.29 ***	0.35 ***	0.44 ***	1				
13. T4 DVW	0.37 ***	0.28 ***	0.16 ***	0.22 ***	0.46 ***	0.32 ***	0.13 ***	0.16 ***	0.36 ***	0.28 ***	0.14 ***	0.19 ***	1			
14. T4 DVV	0.30 ***	0.43 ***	0.20 ***	0.26 ***	0.38 ***	0.48 ***	0.20 ***	0.19 ***	0.27 ***	0.41 ***	0.26 ***	0.24 ***	0.71 ***	1		
15. T4 CDs	0.07	0.14 ***	0.43 ***	0.26 ***	0.10 *	0.17 ***	0.47 ***	0.32 ***	0.14 ***	0.22 ***	0.54 ***	0.36 ***	0.19 ***	0.22 ***	1	
16. T4 BP	0.06	0.12 **	0.23 ***	0.39 ***	0.12 **	0.14 ***	0.29 ***	0.32 ***	0.15 ***	0.23 ***	0.36 ***	0.39 ***	0.19 ***	0.23 ***	0.41 ***	1
*M*	1.33	1.62	2.31	1.30	1.35	1.58	2.19	1.32	1.33	1.47	2.16	1.32	1.33	1.46	2.03	1.25
*SD*	0.62	0.81	0.86	0.53	0.65	0.79	0.84	0.54	0.66	0.76	0.91	0.56	0.70	0.79	0.91	0.51
*Scores range*	1–5		1–6	1–5	1–5		1–6	1–5	1–5		1–6	1–5	1–5		1–6	1–5
*Note.* DVW = domestic violence witnessing; DVV = domestic violence victimization; CDs = cognitive distortions; BP = bullying perpetration. * *p* < 0.05, ** *p* < 0.01, *** *p* < 0.001.																
**(b)**																
	**1**	**2**	**3**	**4**	**5**	**6**	**7**	**8**	**9**	**10**	**11**	**12**	**13**	**14**	**15**	**16**
1. T1 CVW	1															
2. T1 CVV	0.48 ***	1														
3. T1 CDs	0.34 ***	0.21 ***	1													
4. T1 BP	0.33 ***	0.23 ***	0.34 ***	1												
5. T2 CVW	0.45 ***	0.26 ***	0.22 ***	0.29 ***	1											
6. T2 CVV	0.18 ***	0.28 ***	0.10 **	0.16 ***	0.51 ***	1										
7. T2 CDs	0.23 ***	0.15 ***	0.62 ***	0.32 ***	0.33 ***	0.17 ***	1									
8. T2 BP	0.19 ***	0.09 *	0.23 ***	0.37 ***	0.28 ***	0.19 ***	0.33 ***	1								
9. T3 CVW	0.30 ***	0.24 ***	0.11 **	0.20 ***	0.41 ***	0.25 ***	0.18 ***	0.13 ***	1							
10. T3 CVV	0.12 **	0.22 ***	0.09 *	0.12 **	0.16 ***	0.24 ***	0.12 **	0.09 *	0.64 ***	1						
11. T3 CDs	0.22 ***	0.12 ***	0.43 ***	0.23 ***	0.29 ***	0.18 ***	0.53 ***	0.30 ***	0.22 ***	0.16 ***	1					
12. T3 BP	0.20 ***	0.15 ***	0.24 ***	0.29 ***	0.25 ***	0.22 ***	0.34 ***	0.42 ***	0.27 ***	0.25 ***	0.44 ***	1				
13. T4 CVW	0.28 ***	0.16 ***	0.20 ***	0.19 ***	0.36 ***	0.24 ***	0.18 ***	0.17 ***	0.36 ***	0.19 ***	0.24 ***	0.22 ***	1			
14. T4 CVV	0.09 *	0.10 *	0.14 ***	0.13 ***	0.12 **	0.17 ***	0.09 *	0.09 *	0.16 ***	0.23 ***	0.09 *	0.11 **	0.64 ***	1		
15. T4 CDs	0.29 ***	0.14 ***	0.43 ***	0.26 ***	0.19 ***	0.08 *	0.47 ***	0.32 ***	0.14 ***	0.0 7	0.54 ***	0.36 ***	0.26 ***	0.14 ***	1	
16. T4 BP	0.17 ***	0.10 *	0.23 ***	0.39 ***	0.21 ***	0.15 ***	0.29 ***	0.32 ***	0.20 ***	0.11**	0.36 ***	0.39***	0.25 ***	0.13 **	0.41 ***	1
*M*	1.72	1.46	2.31	1.30	1.75	1.40	2.19	1.32	1.65	1.38	2.16	1.32	1.64	1.40	2.03	1.25
*SDs*	0.74	0.62	0.86	0.53	0.80	0.54	0.84	0.54	0.89	0.71	0.91	0.56	0.86	0.77	0.91	0.51
*Scores range*	1–5		1–6	1–5	1–5		1–6	1–5	1–5		1–6	1–5	1–5		1–6	1–5
*Note.* CVW = community violence witnessing; CVV = community violence victimization; CDs = cognitive distortions; BP = bullying perpetration. * *p* < 0.05, ** *p* < 0.01, *** *p* < 0.001.																

**Table 2:** Indirect effects of violence exposure on bullying perpetration via self-serving cognitive distortions (CDs) and reciprocal indirect associations between self-serving CDs and bullying perpetration.

Domestic Violence Exposure			
Indirect Paths	*β* (SE)	95% C.I.s	
		LL	UL
T1 Victimization → T2 CDs → T3 Bullying Perpetration	0.01 * (0.00)	0.002	0.017
T2 Victimization → T3 CDs → T4 Bullying Perpetration	0.01 * (0.00)	0.002	0.018
T1 CDs → T2 Bullying Perpetration → T3 CDs	0.02 *** (0.00)	0.009	0.026
T2 CDs → T3 Bullying Perpetration → T4 CDs	0.02 *** (0.00)	0.008	0.025
T1 Bullying Perpetration → T2 CDs → T3 Bullying Perpetration	0.02 *** (0.01)	0.009	0.028
T2 Bullying Perpetration → T3 CDs → T4 Bullying Perpetration	0.02 *** (0.01)	0.010	0.029
**Community Violence Exposure**			
T1 Witnessing → T2 CDs → T3 Bullying Perpetration	0.01 * (0.00)	0.001	0.018
T2 Witnessing → T3 CDs → T4 Bullying Perpetration	0.01 * (0.01)	0.001	0.020
T1 CDs → T2 Bullying Perpetration → T3 CDs	0.02 *** (0.00)	0.009	0.025
T2 CDs → T3 Bullying Perpetration → T4 CDs	0.02 *** (0.00)	0.008	0.024
T1 Bullying Perpetration → T2 CDs → T3 Bullying Perpetration	0.02 *** (0.01)	0.008	0.026
T2 Bullying Perpetration → T3 CDs → T4 Bullying Perpetration	0.02 *** (0.01)	0.010	0.028

Note. β = standardized estimates; SE = standard error; C.I.s = confidence intervals; LL = lower limit, UL = upper limit; * *p* < 0.05, *** *p* < 0.001.

## Data Availability

Data are available from the corresponding author on reasonable request.

## Supplementary Materials

The following supporting information can be downloaded at: https://www.mdpi.com/article/10.3390/ijerph21070883/s1, Table S1: Participation and attrition rates for each cohort of the study.
