# Supplementary material for: A Four-Wave Cross-Lagged Study of Exposure to Violent Contexts, Cognitive Distortions, and School Bullying during Adolescence
Source: Int J Environ Res Public Health. 2024 Jul 7;21(7):883. doi: 10.3390/ijerph21070883 (PMC11277227; doi:10.3390/ijerph21070883)
Supplement: Supplementary file 1 [file ijerph-21-00883-s001.zip › ijerph-3056042-supplementary.pdf]

Table s1. Participation and attrition rates for each cohort of the study.

	Data points							Participation
	**2013**	**2014**	**2015**	**2016**	**2017**	**2018**	**2019**	**rate**
**1th Cohort**	N = 510	N = 450	N = 407	N = 377				74%
**Participants' dropout**		60 (11.8%)	+ 43 (+ 8.4%)	+ 29 (+ 5.8%)				
**2nd Cohort**				N = 268	N = 257	N = 257	N = 248	93%
**Participants' dropout**					11 (4.1%)	+ 0 (+ 0%)	+ 9 (+ 2.9%)	
